# Effect of In Vitro Digestion on the Antioxidant and Angiotensin-Converting Enzyme Inhibitory Potential of Buffalo Milk Processed Cheddar Cheese

**DOI:** 10.3390/foods10071661

**Published:** 2021-07-19

**Authors:** Amal Shaukat, Muhammad Nadeem, Tahir Mahmood Qureshi, Rabia Kanwal, Muhammad Sultan, Olivier Basole Kashongwe, Redmond R. Shamshiri, Mian Anjum Murtaza

**Affiliations:** 1 Institute of Food Science and Nutrition, University of Sargodha, Sargodha 40100, Pakistan; amal.shaukat@ucp.edu.pk (A.S.); anjum.murtaza@uos.edu.pk (M.A.M.); 2 Institute of Food Science and Technology, University of Central Punjab, Lahore 42000, Pakistan; 3 Department of Food Sciences, Cholistan University of Veterinary & Animal Sciences, Bahawalpur 63100, Pakistan; tahirmahmood@cuvas.edu.pk; 4 Post-Harvest Research Centre, Ayub Agriculture Research Institute, Jhang Road, Faisalabad 38850, Pakistan; rabiak_018@yahoo.com; 5 Department of Agricultural Engineering, Bahauddin Zakariya University, Multan 60800, Pakistan; muhammadsultan@bzu.edu.pk; 6 Leibniz Institute for Agricultural Engineering and Bioeconomy, Max-Eyth-Allee 100, 14469 Potsdam-Bornim, Germany; rshamshiri@atb-potsdam.de

**Keywords:** ACE-inhibition, antioxidant potential, processed cheddar cheese, water-soluble extract, ethanol-soluble extract

## Abstract

The purpose of this study was to develop an in-vitro digestion protocol to evaluate the antioxidant potential of the peptides found in processed cheddar cheese using digestion enzymes. We first studied antioxidant and angiotensin-converting enzyme (ACE) inhibition and antioxidant activities of processed cheddar cheese with the addition of spices e.g., cumin, clove, and black pepper made from buffalo milk and ripened for 9 months. Then we conducted an in vitro digestion of processed cheddar cheese by gastric and duodenal enzymes. Freeze-dried water (WSE) and ethanol-soluble fractions (ESE) of processed cheddar cheese were also monitored for their ACE inhibition activity and antioxidant activities. In our preliminary experiments, different levels of spices (cumin, clove, and black pepper) were tested into a cheese matrix and only one level 0.2 g/100 g (0.2%) based on cheese weight was considered good after sensory evaluation. Findings of the present study revealed that ACE-inhibitory potential was the highest in processed cheese made from buffalo milk with the addition of 0.2% cumin, clove, and black pepper. A significant increase in ACE-inhibition (%) of processed cheddar cheese, as well as its WSE and ESE, was obtained. Lower IC_50_ values were found after duodenal phase digestion compared to oral phase digestion.

## 1. Introduction

Bioactive compounds are “extra nutritional” constituents occurring in foods in small quantities but having health impacts [1]. In milk, bioactive peptides are produced during fermentation with starter cultures (proteolytic), proteolytic enzymatic hydrolysis, and gastrointestinal digestion. Hence, they confer to processed dairy products such as cheese nutritional and health benefits. Cheese could provide antihypertensive peptides devoid of any premeditated functional part [2]. Several studies, concurring with this claim, report associations of dairy products intake with a decrease in blood pressure through specific biological pathways [3]. The rennin-angiotensin-aldosterone system is a biological pathway involved in blood pressure regulation in the human body and affected by bioactive peptides in cheese [4]. The possible health benefits and associations with cheese consumption of these bioactive peptides, resulting from proteolytic digestion of the parent milk proteins, can be monitored. This is possible when considering the natural concentrations of these compounds within food products for example ripened cheese [5]. Antioxidant activity is another important property of bioactive compounds. Superfluous free radicals and oxygen species with high reactivity result in disastrous cellular possessions like apoptosis by cellular proteins (oxidized), enzymes, DNA, and cell membrane lipids. Antioxidants that occur candidly in foods could safeguard the humanoid form by delaying the progress of many chronic diseases [6,7,8].

The functional role of spices used as cheese additives has been reported in many studies. The bark of cinnamon contains “Cinnamaldehyde” as a foremost component with 2% of essential oil. The extract of methanol comprises anthraquinones, tannins, terpenoids, flavonoids, coumarins, and glycosides [9]. Cinnamon contains polyphenolic polymers which were found effective in controlling diabetes and glucose intolerance and also act as antioxidants [10]. The clove oil contains ‘Eugenol’ as a principal component that exhibits significant anticancer, antioxidant, cardiovascular, and anti-inflammatory properties in addition to these, triterpenes, galloyltannins, flavonoids, phenolic acids were also found in clove [11,12]. Compounds e.g., Tannins, phenolics, and flavonoids were found in cumin roots, leaves, and flowers [13]. These pharmacological properties of Nigella sativa oil and seed were due to the occurrence of some essential elements e.g., alpha-hederin, thymoquinone, nigellidine, thymohydroquinone, nigellicine, dithymoquinone, nigellimine-N-oxide, thymol, and carvacrol [14]. The “king of spices” was the name given to black pepper as it was one of the world’s ancient and best-well-notorious spices. Piperine is a major element present in black pepper because of its capability to escalate the digestive capacity by motivating the digestive enzymes of the pancreas which considerably decreases the transit time of food in the gastro-intestinal tract. Black pepper’s essential oil was found to have an anti-microbial property and was able to exhibit inhibitory properties against 25 diverse bacterial categories [15].

A few studies have been conducted on the supplementation of spices in cheese. Ahmed et al. [16] made spicy Mudaffara cheese using clove (*Syzygium aromaticum*), black cumin, and black pepper and monitored their antioxidant activity. In another study, it was reported that the manufacturing of paneer with turmeric powder (turmeric addition at 0.6% proportion by weight of estimated paneer production) enhances its shelf stability fit for 15 days [17]. Josipovic et al. [18] manufactured a novel cottage cheese using spices and found that using pepper in the cheese matrix showed good antimicrobial and antioxidant activity. Few studies were reported regarding the antioxidant potential of spices. To the best of our knowledge previously no research was conducted regarding the effect of in vitro digestion on ACE-inhibitory and antioxidant potential of processed cheddar cheese made from buffalo milk using the spices (Black pepper, clove, and cumin). Furthermore effect of in-vitro digestion was also observed in water and ethanol soluble fractions of processed cheddar cheese. These methods try to mimic physiological evaluation of bioavailability of nutrients in in vivo study needs high cost of the analysis. To study the gastrointestinal behavior of food in vitro digestion methods are now preferably used. This method is more rapid, less expensive, less labor extensive conditions, taking into account the presence of digestive enzymes and their concentrations, pH, digestion time, and salt concentrations, among other factors. In this study, the influence of stimulated in vitro digestion of processed Cheddar cheese (made with the addition of clove, cinnamon, and black pepper) by gastric and duodenal enzymes on the release of bioactive peptides related to antioxidant activity and an antihypertensive activity during ripening was monitored.

## 2. Materials and Methods

### 2.1. Procurement of Raw Material for Cheddar Cheese Manufacturing

Buffalo milk was used for Cheddar cheese manufacturing. Milk (15 L) was collected from the local farm at Sargodha, Pakistan, and standardized at 3.5% fat for cheese manufacturing. All the apparatus was properly cleaned and manufacturing was done under hygienic conditions in the laboratory. All the glassware used were properly washed and sterilized. Chemicals for analysis were purchased from Sigma Aldrich (Seelze, Germany) and Lab-Scan (Dublin, Ireland) available at the local market in Sargodha, Pakistan.

### 2.2. Manufacturing of Processed Cheddar Cheese

The procedure described by Lawrence et al. [19] with some modifications, was adapted to make processed cheddar cheese. After the standardization of milk (15 L), pasteurization was done at 63 °C for 30 min. The active culture was added into the pasteurized milk at 32 °C (slow stirring), and ripening of milk was done for 3 h until the milk pH lowered to 6.4 (before the addition of rennet, pH should be below 6.4 for better rennet activity). To speed up the coagulation, CaCl_2_ (0.05% *w*/*w*) was added to the milk. Then rennet (3.5 mL/15 L) was added into the milk and stirring was stopped. After setting the coagulum (30 min), it was cut to the size of peas. The whey was drained off by pouring in the muslin cloth and after pressing for 15 min, cheddaring was done for 30 min. Then salt (1%) was added into the cheese matrix after milling and mellowing was done for 30 min. The cheese matrix was pressed (2 bar pressure) with a hydraulic press for 60 min. The cheeses were packed and placed in the controlled atmosphere (4 °C) for a ripening period of 9 months.

After melting of 9 months ripened cheeses made within the kettle, three spices namely, cumin, cardamom, and black pepper were added into each cheese. The inclusion levels of the spices were determined in a preliminary experiment where cheeses were rated by their sensory properties and the inclusion level of 0.2% (based on cheese weight) was adopted. During the melting process, some emulsifying salts (phosphates 3%) were also added to avoid fat separation. The mixing of ingredients was done for about 10 min at a temperature of 80–85 °C with steam. The processed cheese was poured into molds and stored at 5 ± 1 °C until further analysis.

### 2.3. Preparation of Freeze-Dried Water Soluble and Ethanol Soluble Extracts (pH 4.6 Soluble Fraction) of Processed Cheddar Cheese

Water-soluble extracts (pH 4.6 soluble fractions) of all the processed cheddar cheese were prepared by the following method of Pripp et al. [20]. The first step was to mix the 15 g of grated cheese in 50 mL water and this mixture was placed in a water bath and heated at 40 °C for 5 min. The second step was a homogenization of the above mixture for 2 min using an Omni-Mixer homogenizer (Omni International, Waterburg, CT). Then 2 M hydrochloric acid was used to adjust the pH at 4.6 and distilled water was added until the sample weight (above grated cheese sample mixture) was equal to 100 g. Again, the samples were placed in a water bath for 1 h at a temperature of 40 °C to soften or melt all the fat in the cheese. Then centrifugation of the samples was done at 4500 rpm (3000× *g*) for 30 min at 40 °C. After centrifugation, filtration was done using Whatman filter paper No. 1. The round bottom flask was used to collect water-soluble fractions or extracts and then were freeze-dried for further analysis. After freeze-drying, powdered samples were weighed and stored at 20 °C in plastic tubes. The remaining 30 mL of supernatant was mixed with 70 mL (*v*/*v* %) of ethanol and retained for 1 h at room temperature. Centrifugation for 10 min was again done to remove precipitates at 4500 rpm (3000× *g*) at a temperature of 20 °C. Supernatant labeled as the ethanol-soluble fraction (ESF) 70% were filtered through Whatman No.1 filter paper. About 60 mL supernatant was collected in a round bottle flask and place separately. Rotator evaporator was used for ethanol removal at 30 °C. Freeze-dried samples were stored at −20 °C until further use.

### 2.4. In Vitro Enzymatic Digestion of Processed Cheddar Cheese

To simulate human digestion in the stomach and the duodenum, an in vitro digestion was performed according to Minekus et al. [21], with some modifications. For the preparation of cheese samples, approximately 1.3 mL simulated salivary fluid (SSF) electrolyte stock solution +5.0 µL of 0.3 M CaCl_2_ was added to shredded cheese samples and mixed properly to get paste-like uniformity. The suggested time of interaction of the sample with the enzyme was 2–3 min done at 37 °C, which required pre-warming of all components to 37 °C. The final volume of oral phase digestion was 2 mL. In the gastric phase, 1.6 mL of stimulated gastric fluid (SGF) stock electrolyte solution was added in the oral bolus or liquid cheese samples to acquire an absolute ratio of samples to SGF 50:50 (*v*/*v*) after addition of water and other recipients. The pepsin solution was prepared by adding 3.71 mg porcine pepsin (EC 3.4.23.1) in 1 mL of distilled water. In the final digestion mixture, porcine pepsin (EC 3.4.23.1) was added to attain 2000 U mL^−1^, followed by 1.0 µL of 0.3 M CaCl_2_ addition to achieve 0.075 mM in the final digestion mixture. Nearly 250 µL of 1 M HCl was required for decreasing the pH of the solution up to 3.0. Lastly, for dilution of the stock solution of SGF, the essential volume of water was added. The measured amount of water was 851 µL to make the final volume of the gastric phase up to 4 mL. The gastric digestion was done for 60 min at 37 °C. After the gastric phase, the final volume of the liquid sample was 4 mL. This 1.6 mL SIF electrolyte solution (stock electrolyte) containing porcine pancreatin was added in gastric samples-chyme. The total of pancreatin added was calculated based on the activity of trypsin (100 UmL^−1^) in the final mixture. The porcine bile in 0.5 mL distilled water. 8.0 µL CaCl_2_ is added to the mixture. To neutralize the mixture to pH 7.0, 100 µL NAOH was added to the digestion mixture. The intestinal digestion was done for 120 min at 37 °C. The intestinal digestion was stopped by adding 96 µL PEFA block. The digested cheese samples were stored in plastic bags at 4 °C for further analysis.

### 2.5. ACE-Inhibition Assay

The ACE-inhibition assay was performed using reversed-phase HPLC according to Qureshi et al. [22], following the method described by Hyun and Shin (2000), with some modifications. The liberated hippuric acid as a result of the reaction between HHL (hippuryl-histidyl-leucine) (substrate) and ACE (enzyme) in the presence or absence (control) of the sample were quantified. The percent ACE inhibition was calculated from the following equation:

$$\mathrm{ACE}\;\mathrm{inhibition}\;\left(\%\right)=\frac{\mathrm{HA}\;\left(\mathrm{control}\right)-\;\mathrm{HA}\;\left(\mathrm{sample}\right)}{\mathrm{HA}\;\left(\mathrm{control}\right)}\times 100$$

where hippuric acid concentration was given as HA (control) and hippuric acid was liberated after reaction between the substrate (without sample) and enzyme, while HA (sample) stands for hippuric acid released after enzyme and substrate (presence of sample) reaction.

### 2.6. Determination of Total Phenolic Content of Processed Cheddar Cheese

The total Phenolics contents were determined by using the Folin–Ciocalteu reagent method with some modifications [23]. The freeze-dried samples of cheese samples were dissolved in ethanol and then filtered through 0.45 μm filters. The 0.5 mL sample was mixed with 1 mL of Folin–Ciocalteu reagent (10%). Then 2 mL of sodium carbonate (20%) solution was added into the above mixture after 6 min. The absorbance was taken at 760 nm using a spectrophotometer after 60 min incubation at 30 °C.

### 2.7. Determination of Total Antioxidant Capacity (TAC) of Processed Cheddar Cheese

For the determination of total antioxidant capacity (TAC), the WSE and ESE of cheese samples were analyzed using the method described by Prieto et al. [24]. The freeze-dried cheese samples were dissolved in ethanol and then were filtered through 0.45 μm filters. 0.4 mL of sample was mixed with 4 mL of reagent (0.6 M sulfuric acid, 28 mM sodium phosphate, and 4 mM ammonium molybdate) solution. After incubating the mixture for 95 min at 90 °C, the absorbance was measured at 695 nm using a spectrophotometer. The spectrophotometer was calibrated with blank (methanol) solution prepared in the same manner.

### 2.8. Determination of DPPH Radical Scavenging Activity of Processed Cheddar Cheese

Using the method prescribed by Yi et al. [25] with some modifications, the capability of WSE and ESE of all the cheeses to scavenge 2, 2-diphenyl-1-picrylhydrazyl radical (DPPH) was measured. The ethanol was used to dissolve the freeze-dried samples of cheese and then filtration (0.45 μm filters) was carried out before running the DPPH assay. 1 mL of water-soluble extract (WSE) and ethanol-soluble extract (ESE) were taken and then added 2 mL of DPPH (60 μm in ethanol) solution into each separately. Then incubation was done for 30 min in the dark. The spectrophotometer was used to measure the absorbance at 517 nm. The preparation of control (ethanol) was also done using the same method.

### 2.9. Reducing Power Ability of Processed Cheddar Cheese

The method described by Hegazy and Ibrahium [26] was adopted with some modifications to determine the reducing power of WSE and ESE of cheese samples. The freeze-dried samples of cheese were dissolved in ethanol and then filtered through 0.45 μm filters. Each 0.5 mL sample was mixed with 0.5 mL sodium phosphate buffer (0.2 M, pH 6.6) and potassium ferricyanide (K_3_Fe (CN_6_)). After incubating the mixture for 20 min (50 °C), 0.5 mL of trichloroacetic acid (%) was added to it. After 15 min, 0.2 mL of ferric chloride (0.1%) was added. The absorbance of the sample mixture was measured using a spectrophotometer at 700 nm.

### 2.10. Sensory Evaluation

The score for overall acceptability, appearance, flavor, taste, texture, and color for cheese samples was evaluated using 9 point hedonic scale. The sensory evaluation of cheese samples was done by a panel of 15 judges including faculty members and students at the Institute of Food Science and Nutrition, University of Sargodha, Sargodha.

### 2.11. Statistical Analysis

Statistical analysis was performed using Minitab 16 software. A 3-factors factorial design was executed with repeat batches (random variable), age (static variable; with the supposition that the individual cheeses were measured independently from the same batch), and milk type (fixed variable) of Cheddar cheese. Similarly, 3-factors factorial design was carried out with replicate block (random variable), digestion steps (fixed variable; including undigested samples), and milk type (fixed variable) of Cheddar cheese. Tukey test for pair-wise comparison was used to test the differences between means. For all comparisons, the level of significance was set to *p* < 0.05.

## 3. Results

### 3.1. Total Antioxidant Activity (TAA) of Processed Cheddar Cheese (PCS), Orally Digested (PODC), and Duodenal Digested Cheddar Cheese (PDC)

#### Total Antioxidant Activity (TAA) of Freeze-Dried Water Soluble and Ethanol Soluble Extract of Processed Cheddar Cheese

**Figure 1:**
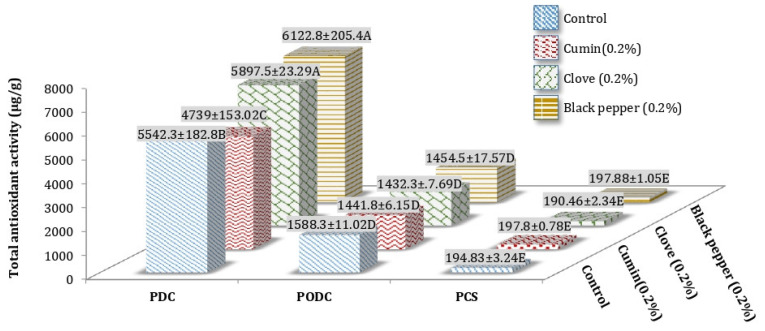
Mean values for Total antioxidant activity (TAA) of processed Cheddar cheese (PCS), orally digested (PODC), and duodenal digested Cheddar cheese (PDC) made from buffalo milk after ripening. A-E means followed by the same letter for the same parameter are not significantly different (*p* < 0.05).

The processed cheddar cheese with black pepper (6122.8 ± 205.4 µg/g Trolox equivalent) and clove (5897.5 ± 23.29 µg/g Trolox equivalent) powder shows the maximum values for TAA after in vitro digestion of ripened (PDC), while the minimum TAA was observed in case of cumin processed Cheddar cheese (4739 ± 153.02 µg/g Trolox equivalent) (Figure 1).

**Figure 2:**
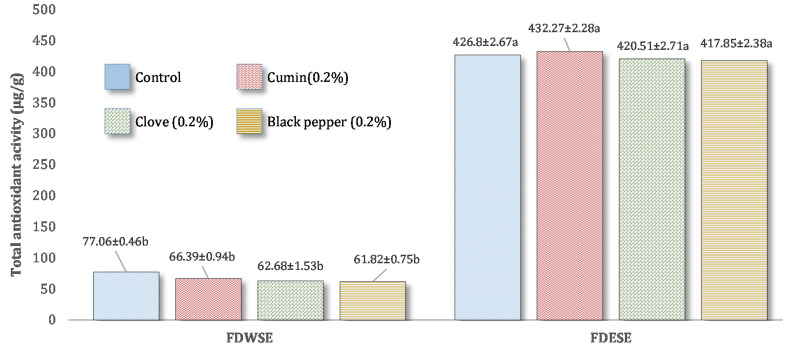
Mean values for Total atioxidant activity (TAA) of freeze-dried water-soluble (WSE) and ethanol-soluble extract (ESE) of processed Cheddar cheese made from buffalo milk after ripening. a,b means followed by the same letter for each type of processed Cheddar cheese are not significantly different (*p* < 0.05).

Freeze-dried ethanol extract of processed Cheddar cheese showed increased total antioxidative potential with the addition of spices such as cumin, clove, and black pepper powder with 0.2% concentration as compared to water-soluble extract. This may be because peptides which are responsible for antioxidative activity were more soluble in ethanol as compared to water. Among the spices, the higher total antioxidant capacity (432.27 ± 2.28 µg/g Trolox equivalent) was observed in ethanol extract of cumin processed cheddar cheese as compared to water-soluble extract (Figure 2). The processed cheddar cheese extracts (WSE and ESE) from cow and buffalo milk have greater potential for exhibiting total antioxidant potential as compared to control cheddar cheese extracts (WSE and ESE). Thus, this may be due to the antioxidative potential of individual spices as reported by different studies.

### 3.2. Total Phenolic Content (TPC) of Processed Cheddar Cheese (PCS), Orally Digested (PODC), and Duodenal Digested Cheddar Cheese (PDC)

**Figure 3:**
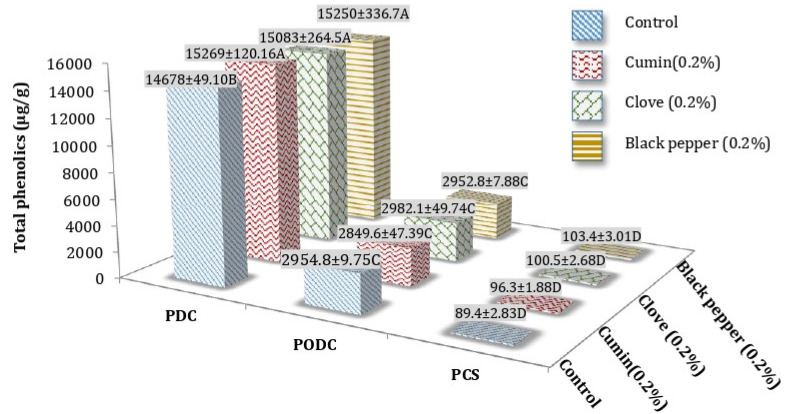
Mean values for Total phenolic content (TPC) (µg/g gallic acid equivalent) of processed cheddar cheese (PCS), orally digested (PODC), and duodenal digested cheddar cheese (PDC) made from buffalo milk after ripening. A-D means followed by the same letter for the same parameter are not significantly different (*p* < 0.05).

The TPC of processed Cheddar cheese increased after oral and duodenal stimulated in vitro digestion, although no considerable effect of individual spices was observed on TPC of cheese after digestion. After in vitro digestion at 9 months of ripening, clove PODC (2982.1 ± 4.74 µg/g gallic acid equivalent) and cumin PDC (15269 ± 1.16 µg/g gallic acid equivalent) showed the maximum mean values (Figure 3). No significant increase in phenolic content of ripened processed cheddar cheese was observed with the addition of spices before and after digestion.

#### Total Phenolic Content (TPC) of Freeze-Dried Water Soluble and Ethanol Soluble Extract of Processed Cheddar Cheese xxx

**Figure 4:**
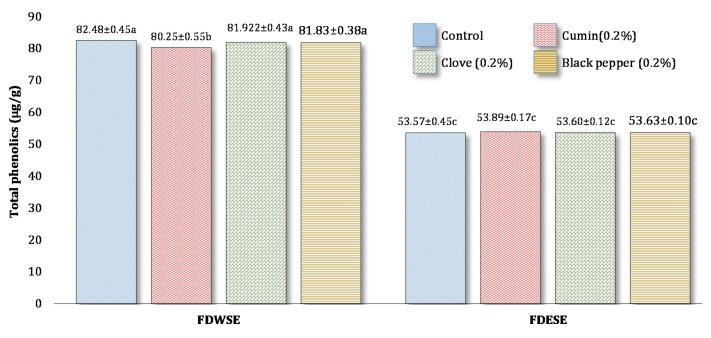
Mean values for Total phenolic content (TPC) (µg/g gallic acid equivalent) of freeze-dried water-soluble (WSE) and ethanol-soluble extract (ESE) of processed Cheddar cheese made from buffalo milk after ripening. a-c means followed by the same letter for each type of processed Cheddar cheese are not significantly different (*p* < 0.05).

The phenolic content of WSE and ESE of processed cheese was not affected by the addition of spices. Higher phenolic content values were observed in water-soluble extracts as compared to ethanol-soluble extract (Figure 4). Spices were not found to contain any phenolic compounds. More studies need to be done to find their effect on the antioxidant potential of cheese.

### 3.3. Reducing Power (RP) of Processed Cheddar Cheese (PCS), Orally Digested (PODC), and Duodenal Digested Cheddar Cheese (PDC)

**Figure 5:**
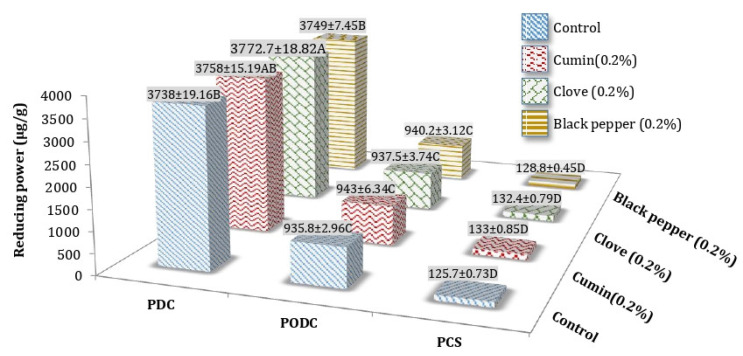
Mean values for Reducing power (RP) (µg/g Trolox equivalent) of processed Cheddar cheese (PCS), orally digested (PODC), and duodenal digested Cheddar cheese (PDC) made from buffalo milk after ripening. A-D means followed by the same letter for the same parameter are not significantly different (*p* < 0.05).

A significant increase in reducing power ability of ripened processed Cheddar cheese was observed with the addition of spices before and after digestion. The RP of processed Cheddar cheese increased after oral and duodenal stimulated in vitro digestion, although the effect of individual spices was observed on RP of cheese after digestion might be due to the release of compounds from cheese and spices which have redox potential. After in vitro oral and duodenal digestion at 9 months of ripening cumin PODC (943 ± 6.34 µg/g Trolox equivalent) and clove PDC (3772.7 ± 8.82 µg/g Trolox equivalent) showed the maximum mean values respectively (Figure 5).

#### Reducing Power (RP) of Freeze-Dried Water-Soluble (WSE) and Ethanol Soluble Extract (ESE) of Processed Cheddar Cheese

**Figure 6:**
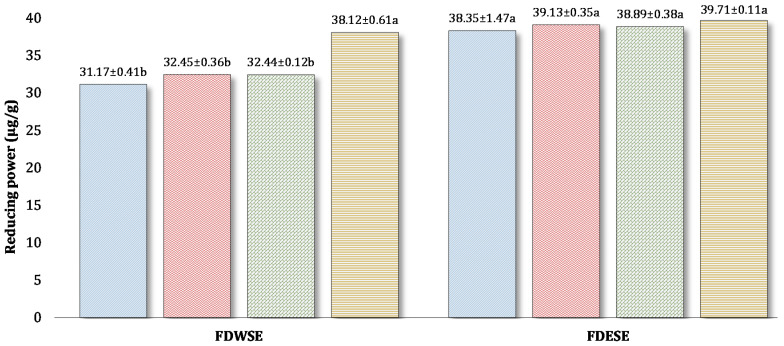
Mean values for Reducing power (RP) (µg/g Trolox equivalent) of freeze-dried water-soluble and ethanol-soluble extract of processed Cheddar cheese made from buffalo milk after ripening. a-b means followed by the same letter for each type of processed Cheddar cheese are not significantly different (*p* < 0.05).

Spices were found to contain limited compounds which exhibit reducing power, however, processing and storage conditions might be responsible for reduced RP in WSE and ESE of processed cheddar cheese. The highest reducing power was observed in black pepper cheese WSE (38.12 ± 0.61 µg/g Trolox equivalent) and black pepper cheese ESE (39.71 ± 0.11 µg/g Trolox equivalent) after ripening in buffalo processed cheddar cheese (Figure 6).

### 3.4. DPPH (Radical Scavenging Activity) of Processed Cheddar Cheese (PCS), Orally Digested (PODC), and Duodenal Digested Cheddar Cheese (PDC)

**Figure 7:**
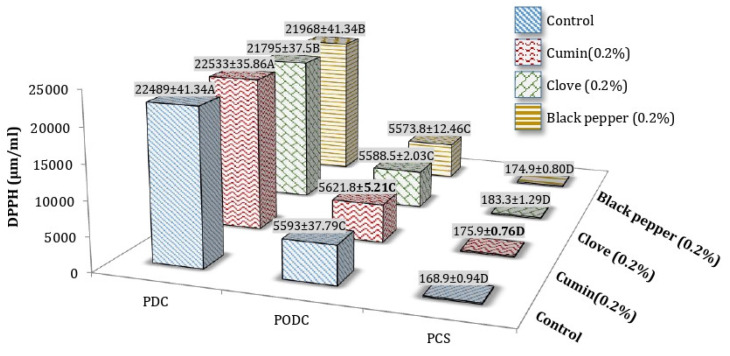
Mean values for DPPH (Radical Scavenging Activity) (µmol/mL Trolox equivalent) of processed Cheddar cheese (PCS), orally digested (PODC) and duodenal digested Cheddar cheese (PDC) made from buffalo milk after ripening. A-D means followed by the same letter for the same parameter are not significantly different (*p* < 0.05).

A substantial increase in radical scavenging activity (DPPH) of ripened processed Cheddar cheese was observed with the addition of spices before and after digestion. The DPPH of processed Cheddar cheese increased after oral and duodenal stimulated in vitro digestion, although the effect of individual spices was also observed on DPPH of cheese after digestion might be due to the release of compounds from cheese and spices which have peptides with radical scavenging ability. Among processed Cheddar cheese samples, radical scavenging activity of cheese was affected by the addition of spices (cumin, clove, and black pepper). After in vitro digestion at 9 months of ripening, cumin PODC (5621.8 ± 5.21 µmol/mL Trolox equivalent) and cumin PDC (22,533 ± 3.86 µmol/mL Trolox equivalent) showed the maximum mean values (Figure 7).

#### DPPH (Radical Scavenging Activity) of Freeze-Dried Water-Soluble (WSE) and Ethanol Soluble Extract (ESE) of Processed Cheddar Cheese

**Figure 8:**
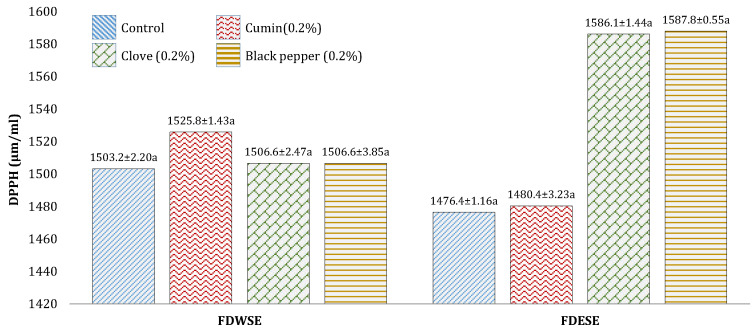
Mean values for DPPH (Radical Scavenging Activity) (µmol/mL Trolox equivalent) of freeze-dried water-soluble and ethanol-soluble extract of processed Cheddar cheese made from buffalo milk after ripening. a means followed by the same letter for each type of processed Cheddar cheese are not significantly different (*p* < 0.05).

The highest DPPH value was observed in cumin cheese WSE (1525.8 ± 1.43 µmol/mL Trolox equivalent). In case of ethanol-soluble extract, black pepper cheese (1587.8 ± 0.55 µmol/mL Trolox equivalent) and clove (1586.1 ± 1.44 µmol/mL Trolox equivalent) showed maximum values (Figure 8).

### 3.5. Determination of ACE-Inhibition (%) and IC_50_ Values (mg/mL) of Processed Cheddar Cheese

The inhibitory activity of angiotensin 1-converting enzyme (ACE) was estimated after in vitro oral and duodenal enzymatic digestion in buffalo milk processed Cheddar cheese (addition of spices e.g., cumin, clove, and black pepper at 0.2 g/100 g concentration) after ripening. The inhibitory activity of ACE was also monitored in water-soluble extract (WSE) and ethanol-soluble extract (ESE) of processed Cheddar cheese. ACE inhibitory activity is expressed as IC_50_ value (as the amount of protein content needed to inhibit 50% of the ACE inhibitors). In 9 months ripened processed Cheddar cheese (PODC) with the addition of spices e.g., cumin (CPODC), clove (LPODC), black pepper (BPPODC) after oral digestion the maximum value for ACE-inhibition % was observed in BPPODC 69.1 ± 0.55% (IC_50_ value 0.76 ± 0.01 mg/mL) (Table 1). The duodenal digestion significantly increases the percent inhibitory activity of ACE in Cheddar cheese with ripening which may be resulted from the production of more ACE inhibitory peptides after degradation of proteins in in vitro digestion. After duodenal digestion the maximum value for ACE-inhibition % was observed in black pepper BPPDC 91.2 ± 0.75% (IC_50_ value 0.56 ± 0.01 mg/mL) (Table 1).

#### Determination of ACE-Inhibition % and IC_50_ (mg/mL) Values of Freeze-Dried Water (WSE) and Ethanol Soluble Extract (ESE) of Processed Cheddar Cheese

Lower IC_50_ values were obtained for freeze-dried ESE as compared to WSE. More ACE inhibitory peptides may be present in ESE with more potent ACE inhibition activity as compared to WSE. The highest ACE inhibition percentage was found in BPFDESE 90.3 ± 0.49% (IC_50_ value 0.19 ± 0.02 mg/mL) while in freeze-dried water-soluble extract more percentage was found in BPFDWSE 88.4 ± 0.32% (IC_50_ value 0.21 ± 0.01 mg/mL) (Table 2). Lower IC_50_ values were calculated in freeze-dried ESE of black pepper processed cheddar cheese as compared to WSE after a ripening period of 9 months (Table 2).

### 3.6. Sensory Score of Processed Cheddar Cheese

**Figure 9:**
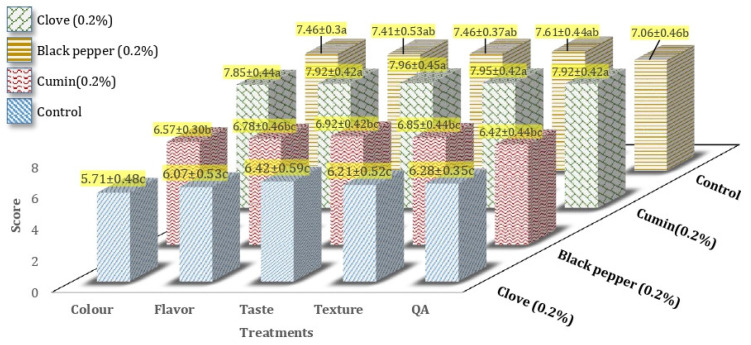
Mean values for a sensory score of processed Cheddar cheese made from buffalo milk after storage. a-c means followed by the same letter for the same parameter are not significantly different (*p* < 0.05).

All the cheese samples of processed Cheddar cheese (after-ripening) were subjected to sensory evaluation with a panel of fifteen judges and evaluated for different sensory attributes like flavor, taste, color, texture, and overall acceptability, following the 9 Point Hedonic Scale Performa presented to the panelists for recording scores. Highly significant (*p* ˂ 0.01) effect of spices was observed on the sensory score of color, flavor, taste, texture, and overall acceptability of processed cheddar cheese (buffalo milk) after ripening. The best score for color (7.85 ± 0.44), flavor (7.92 ± 0.42), taste (7.96 ± 0.45) and texture (7.95 ± 0.42), overall acceptability (7.92 ± 0.42) were observed for cumin processed cheddar cheese after-ripening (Figure 9). The development of flavoring compounds was improved with the progress of ripening. It means ripening positively affected the taste, texture, color, and overall acceptability of processed Cheddar cheese made from buffalo milk.

## 4. Discussion

The results obtained regarding ACE-inhibitory activity after digestion of processed cheese using intestinal enzymes in the present study were concurrent to the studies conducted by Qureshi et al. [22] Srinivas et al. [27]. In addition, Srinivas et al. [27] and Barac et al. [28] also found increased antioxidant activity after digestion of bovine milk proteins and Serbian white-brined cheese respectively, using enzymes which is consistent with our results of the present study. No doubt, many bioactive peptides from milk proteins are also released during the gastric phase [22,29] but in the present study, we conducted oral phase and intestinal phase digestion experiments because most of the bioactive peptides are liberated in the duodenal phase compared to the gastric phase [22].

The peptides in the intact condition of original proteins are usually inactive within the sequence but can be liberated through the action of different enzymes from the stomach (especially pepsin), intestine (chymotrypsin and others) in in vitro models [30,31]. The liberated peptides through gastrointestinal digestion are active and have been shown different bioactivities like for instance, ACE-inhibitory and antioxidant activities, in different types of cheeses [32,33,34,35]. The increased bioactivities after simulated gastrointestinal digestion might be due to the liberation of more and more low molecular weight peptides in the extracts.

Scarce data is available on the ACE-inhibitory and antioxidant activities of cheese with spices, to compare with our studies but Ahmed et al. [16] investigated the antioxidant potential of plaited pickled cheese (Mudaffara) cheese with the addition of black pepper, black cumin. Both ACE-inhibitory and antioxidant activities of different types of cheeses increased with the progress of the ripening period [36,37,38,39] which corroborate the results obtained in the present study. In some previous studies, it was found that most of the bioactive peptides are dissolved in ethanol-soluble fractions of cheeses and these fractions showed potent bioactivities compared to water-soluble fractions [20]. Therefore, in the present study, both WSE and ESE were studied.

The results of TAA of processed cheddar cheese after in-vitro digestion results were similar to those by Lee et al. [40] who made soft goat cheese (unfrozen and 3 months frozen) with the addition of tocopherol. Ahmed et al. [16] determined the antioxidant activity of individual spices such as black cumin (*Nigella sativa*), clove (*Syzygium aromaticum*), and black pepper (*Piper nigrum*) using (DPPH free radical scavenging assay). The Mudaffara cheese was prepared using the spices and its sensory evaluation and antioxidative potential were measured [16]. According to research, the highest antioxidative potential of ethanol and water-soluble extracts of black pepper were determined and confirmed [41,42,43]. As well as highest antioxidation potential was estimated and observed in clove. The extract obtained from clove was reported to have the strongest antioxidative capacity which may be due to its greater metal chelating ability and hydrogen bonding capacity and additionally its efficiency was due to the presence of free radicals, superoxide, and may act as a scavenger of hydrogen peroxide [44]. Increase values for TPC were obtained in digested cheese samples (fortified with an increased amount of catechins) while the value for TPC was measured double as compared to the value of control in the case of cheese fortified with 500 mg/kg catechin [45]. Various varieties of cheeses were investigated for their total phenolic content [46] e.g., smoked cheeses, goat milk cheese (where animals feed on different feeding systems), and production of cheeses from milk using different quantities of plant extracts [47,48]. According to Levkov et al. [49], no survey was performed regarding the phenolics content of conventionally manufactured cheeses and the potential of these compounds to hinder the oxidation process.

The results of RP were in accordance with the studies done by Liu et al. [50]. The results were reported to significantly increase (*p* < 0.05) antioxidant activity after simulated gastrointestinal digestion. The reducing power values for Cheddar cheese samples B-1 (Cheddar cheese with starter culture) and B-2 (Cheddar cheese with starter culture and Lactobacillus rhamnosus) were 0.503 and 0.696 respectively which signifies an increase of 13.03 (%) and 17.57 (%) individually [50]. The results were in accordance with the finding by Abadíagarcía et al. [51]. According to research, extension in the ripening period of cheddar cheese might result in enhanced antioxidation potential due to the production of peptides that exhibit antioxidant potential [51]. The contribution ability of samples for protons and electrons was dependent on the relation of peptide cleavages and reducing power [52]. The finding of DPPH was supported by studies of Ahmed et al. [16]. It was confirmed by the studies that the addition of 0.5% black pepper (grounded form) during manufacturing significantly (*p* ≥ 0.05) raised the antioxidant potential of Mudaffara cheese (65.3%) as compared to fresh cheese (48.4%) as well as the amount of tested ethanol (extract) increases from 50 to 200 μg. The concentrations of ethanol extract have a considerable positive impact on the antioxidative potential of cheese with a ripening period of 4 or 8 weeks at 30 ± 2 or 7 ± 2 °C temperature, respectively. These results were confirmed by Burits and Bucar [53]. According to research, it was confirmed that peptides were present in WSE which were easily reachable to DPPH as well as Hydroxyl radicals [54]. The higher mean values for DPPH peptides were observed after 60 days of ripening but a decreasing trend was found after 120 days of ripening. According to another research, the antioxidative potential of Cheddar cheese was observed higher in the first ripening period while decreased in 2nd period of ripening [55]. Seed (Piper nigrum Linn.) of black pepper along with its WSE and ESE were reported to exhibit antioxidant as well as (DPPH) radical scavenging activities [15,56,57].

The results of ACE inhibition after in-vitro digestion were in close agreement with Qureshi et al. [58]. According to research, it was reported that higher values for ACE inhibition were obtained in Gamalost (Norwegian cheese) which confirmed the production of more peptides (with potent ACE inhibitory potential) after gastrointestinal digestion. More peptides were generated after digestion with human gastric juice and duodenal gastric juice which possesses additional activity as equated to undigested peptides, this resulted in lower IC_50_ values. The peptides which release after gastrointestinal digestion exhibited greater ACE inhibitory potential and possess molecular mass less than 3kDa in Norvegia cheese and soluble fraction of Gamalost (pH 4.6); as the noticeable decrease in IC_50_ values was obtained after gastrointestinal digestion [58]. The results of ACE inhibition potential of water-soluble and ethanol-soluble fractions were similar to the study conducted by Bara’ et al. [28]. It was reported that water-soluble fractions of traditional cheeses exhibited different potential for ACE inhibition. The water-soluble fractions vary with variety and their IC_50_ values were from 2.26 to 4.61 (mg/mL). Generally, lower ACE inhibition activity was observed in sheep milk cheeses as compared to cow cheeses. The good ACE inhibitory potential was measured in WSF of cow cheese (Zlatar) while WSF of sheep cheese (Sjenica) possesses the lowest ACE inhibition potential.

The results regarding the change in color of cheese are similar to the findings of Mamo [59]. A significant change (*p* < 0.05) in the color of cheese was obtained within the ripening period. It was observed that color changes positively affect the sensory score of cheese as the color gets better with ripening. The mean values for color score increased from 7.15 to 7.95 during ripening. Milk constituents, manufacturing approaches and ripening state may prejudice the color of cheese, while the cheese color varies with the variety of cheese and it has no adverse effect on cheese flavor [60]. The results of the flavor score are like the findings of Mamo [59]. It was reported that ripening significantly (*p* < 0.05) affected the flavor of the cheese. The flavor of cheese improved with ripening and the mean values for flavor ranged from 8.0 (ripened) to 4.60 (pre-ripened). Many compounds were responsible for flavor characterization of cheese e.g., sequence of microbial, metabolic, and biochemical alterations during cheese ripening as well as enzymes (milk and rennet), starter bacteria, lipases and microflora (secondary) may contribute to flavor development in cheese. Mamo et al. [59] observed a significant difference in the taste score of cheese samples with ripening. Ripened cheese tasted much well with a mean value for taste score of 8.15 as compared to pre-ripened cheese samples with mean values for taste score of 5.80 [59]. It was identified that sodium chloride was added during cheese preparation was responsible for the salty taste of cheddar cheese due to its organic ions. Content of FAA and peptides stimulated the umami, bitter and sweet taste in cheddar cheese [61]. Mamo et al. [59] reported a positive increase in texture score of cheese with ripening. According to Murtaza et al. [62], different biochemical and microbial alterations that occurred throughout ripening were responsible for texture changes and development. Mamo et al. [59] observed that the overall acceptability score was higher in ripened cheese (8.05) as compared to pre-ripened cheese (6.15). According to Ahmed et al. [16], spices affected the overall acceptability score of cheese (Mudaffara) during storage (room and refrigeration temperature). The black pepper cheese (19.0) and black cumin cheese (18.4) samples at 7 ± 2 °C temperature were found with more overall acceptability score during ripening (after 4th week) whereas, the overall acceptability score for clove cheese was higher after 2nd week of ripening.

## 5. Conclusions

This study showed that the ACE-inhibitory potential was highest in processed cheese (with the addition of 0.2% cumin, clove, and black pepper) made from buffalo milk. A higher ACE-Inhibition percentage was observed in the ethanol-soluble extract as compared to the water-soluble extract of processed cheddar cheese. Among the spices, a higher ACE-Inhibition percentage was found in black pepper processed cheddar cheese after ripening as compared to cumin and clove. The antioxidant activity was increased after in vitro digestion in processed cheddar cheese. The addition of spices also considerably increases the antioxidant potential of ripened processed cheddar cheese before and after in vitro digestion.

## Figures and Tables

**Table 1:** ACE inhibitory activity is expressed as IC_50_ value (as the amount of protein content needed to inhibit 50% of the ACE inhibitors).

Cheddar Cheese Samples	Treatments	ACE-Inhibition Activity	
		ACE-Inhibition (%)	IC_50_ Values mg/mL
Orally Digested processed cheddar cheese samples	CPODC LPODC BPPODC	66.2 ^c^ ± 1.00 67.5 ^b^ ± 0.47 69.1 ^a^ ± 0.55	0.95 ^a^ ± 0.01 0.86 ^b^ ± 0.01 0.76 ^c^ ± 0.01
Duodenal digested processed cheddar cheese samples	CPDC LPDC BPPDC	89.5 ^b^ ± 0.55 90.3 ^ab^ ± 0.33 91.2 ^a^ ± 0.75	0.75 ^a^ ± 0.03 0.67 ^b^ ± 0.01 0.56 ^c^ ± 0.01

CPODC: Orally digested processed Cheddar cheese sample (addition of cumin powder 0.2% after-ripening). LPODC: Orally digested processed Cheddar cheese sample (addition of clove powder 0.2% after-ripening). BPPODC: Orally digested processed Cheddar cheese sample (addition of black pepper powder 0.2% after-ripening). CPDC: Digested processed Cheddar cheese sample (addition of cumin powder 0.2% after-ripening). LPDC: Digested processed Cheddar cheese sample (addition of clove powder 0.2% after-ripening). BPPDC: Digested processed Cheddar cheese sample (addition of black pepper powder 0.2% after-ripening). Each observation is a mean of three replicates of each batch. Results are expressed as mean of scores ± standard error of mean. Means sharing same letters are statistically non-significant (*p* ˃ 0.05).

**Table 2:** ACE inhibitory activity is expressed as IC_50_ value (as the amount of protein content needed to inhibit 50% of the ACE inhibitors).

	Cheddar Cheese Samples (Buffalo Milk)	ACE-Inhibition Activity	
		ACE-Inhibition (%)	IC_50_ Values mg/mL
Water Soluble Extract of processed cheddar cheese samples	CFDWSE	82.3 ^e^ ± 0.47	0.26 ^a^ ± 0.01
	LFDWSE	85.5 ^cd^ ± 0.43	0.24 ^b^ ± 0.02
	BPFDWSE	88.4 ^b^ ± 0.32	0.21 ^e^ ± 0.01
Ethanol Soluble extract of processed cheddar cheese	CFDESE	84.1 ^de^ ± 0.43	0.23 ^c^ ± 0.01
	LFDESE	87.2 ^bc^ ± 0.33	0.22 ^d^ ± 0.03
	BPFDESE	90.3 ^a^ ± 0.49	0.19 ^f^ ± 0.02

CFDWSE: Freeze-dried water-soluble extract cumin cheese sample (Cumin 0.2%). LFDWSE: Freeze-dried water-soluble extract clove cheese sample (Clove 0.2%). BPFDWSE: Freeze-dried water-soluble extract BP cheese samples (Black pepper 0.2%). CFDESE: Freeze-dried ethanol-soluble extract cumin cheese sample (Cumin 0.2%). LFDESE: Freeze-dried ethanol-soluble extract clove cheese sample (Clove 0.2%). BPFDESE: Freeze-dried ethanol-soluble extract BP cheese samples (Black pepper 0.2%). Each observation is a mean of three replicates of each batch. Results are expressed as mean of scores ± standard error of the mean. Means sharing same letters are statistically non-significant (*p* ˃ 0.05).

## Data Availability

Data is contained within the article.
